# Expression of area-specific M2-macrophage phenotype by recruited rat monocytes in duct-ligation pancreatitis

**DOI:** 10.1007/s00418-016-1406-y

**Published:** 2016-02-09

**Authors:** Enqiao Yu, Mataro Goto, Hisashi Ueta, Yusuke Kitazawa, Yasushi Sawanobori, Taro Kariya, Masaru Sasaki, Kenjiro Matsuno

**Affiliations:** Department of Anatomy (Macro), School of Medicine, Dokkyo Medical University, Mibu, Tochigi 321-0293 Japan; Department of General Surgery, Affiliated Wujiang Hospital of Nantong University, Suzhou, Jiangsu China; NHO Miyakonojo Medical Center, Miyazaki, Japan

**Keywords:** Pancreatitis, Multicolor immunostaining, Cellular kinetics, Proliferation, Monocyte recruitment, M2 macrophage

## Abstract

Acute pancreatitis remains a disease of uncertain pathogenesis and no established specific therapy. Previously, we found a predominant increase and active proliferation of macrophages in the inflamed tissues of a rat duct-ligation pancreatitis model. To analyze the origin and possible role of these macrophages, we investigated their in situ cellular kinetics in a rat model of duct-ligation pancreatitis using a recently established method of multicolor immunostaining for macrophage markers and for proliferating cells with ethynyl deoxyuridine. To detect monocyte-derived macrophages, green fluorescent protein-transgenic (GFP^+^) leukocytes were transferred to monocyte-depleted recipients. In the inflamed pancreas, infiltrating macrophages were mainly two phenotypes, CD68^+^CD163^−^ round cells and CD68^+^CD163^+^ large polygonal cells, both of which showed active proliferation. In the interlobular area, the proportions of CD68^+^CD163^low^ and CD68^+^CD163^high^ cells increased over time. Most expressed the M2-macrophage markers CD206 and arginase 1. In contrast, in the interacinar area, CD68^+^ cells did not upregulate CD163 and CD206, but ~30 % of them expressed the M1 marker nitric oxide synthase 2 on day 4. GFP^+^-recruited cells were primarily CD68^+^CD163^−^ monocytes on day 1 and showed phenotypic changes similar to those of the monocyte non-depleted groups. In conclusion, infiltrating macrophages mostly formed two distinct subpopulations in different areas: monocyte-derived macrophages with the M2 phenotype in the interlobular area or non-M2 phenotype in the interacinar area. Involvement of resident macrophages might be minor in this model. These results are the first demonstration of an upregulated M2 phenotype in rat inflammatory monocytes, which may promote tissue repair.

**Electronic supplementary material:**

The online version of this article (doi:10.1007/s00418-016-1406-y) contains supplementary material, which is available to authorized users.

## Introduction

Acute pancreatitis remains a disease of uncertain pathogenesis and no established specific therapy. Although this inflammation is associated with persistent destruction of the acinar cells, some repair processes must occur as well. Previously, employing a rat duct-ligation pancreatitis model, we found that the majority of infiltrating cells were macrophages (Yamaguchi et al. 1993). 5-bromo-2′-deoxyuridine (BrdU) immunostaining revealed active proliferation of duct epithelia on days 1–2 and proliferation of acinar cells on days 2–28, although acinar cells showed progressive atrophy.

Furthermore, with additional BrdU immunostaining, infiltrating cells with different rat macrophage markers, such as CD68 (detected by ED1 monoclonal antibody), CD163 (ED2), CD11b/c (OX42), and class II major histocompatibility complex molecule (MHCII), showed active proliferation with a peak on day 2 after duct-ligation surgery (Goto et al. 1993). ED1 recognizes a lysosomal protein and is considered the rat equivalent of CD68 (Damoiseaux et al. 1994). CD163 is a haptoglobin receptor expressed on tissue macrophages (Polfliet et al. 2006). CD11b/c is expressed by monocytes, a subset of macrophages, and dendritic cells (Yu et al. 2012). Macrophages upregulate MHCII when activated by cytokines such as IFN-γ (Stet et al. 1986). These macrophages might be derived from monocytes or resident macrophages, as has been suggested (Goto et al. 1993); however, their exact origin and roles in tissue repair or destruction processes have not been studied yet.

Several macrophage subsets with distinct functions are well known (Murray and Wynn 2011). Classically activated macrophages (M1 macrophages) mediate defense of the host from a variety of microbes and promote pro-inflammatory responses with tissue damage. Alternatively activated macrophages (M2 macrophages) have an anti-inflammatory function and regulate wound healing. Because CD163 is an M2-related marker in humans (Komohara et al. 2006) and possibly rats (Cote et al. 2013), we suspected that proliferating CD163^+^ cells might correspond to M2 macrophages.

Answering these questions requires an appropriate technique for tracking different macrophages and multicolor immunohistological staining for detecting several different markers on a single cell surface. For cell tracking, green fluorescent protein-transgenic (GFP^+^) rats are now available and suitable for cell transfer studies of the monocyte lineage. For immunohistological staining, enzyme-developed color dyes tend to interfere with the subsequent immunostaining step, and detection of two different markers on a single cell surface has been very difficult; at best, only two-color analysis prior to BrdU staining could be performed (Matsuno et al. 2010).

Recently, we developed a new method of multicolor immunofluorescence staining for tissue sections using a thymidine analog, 5-ethynyl-2′-deoxyuridine (EdU) (Kitazawa et al. 2015). Because of the small size of azide dye, using click chemistry and elimination of DNA denaturation steps, EdU staining allows for immunofluorescence staining of at least four colors, including two different markers on a single cell surface, which was impossible with the standard BrdU method.

In the present study, we performed immunohistological analysis of macrophages in this pancreatitis model by using multicolor immunostaining methods, including EdU. In some rats, monocytes were partially depleted and received GFP^+^ monocytes intravenously.

## Materials and methods

### Animals and surgical procedures

Male outbred Wistar rats, age 3–5 months, and inbred male Lewis rats (RT1A^l^B^l^), age ~3 months, were supplied by the Laboratory Animal Center for Experimental Research, Kumamoto University School of Medicine and by SLC Co. (Shizuoka, Japan), respectively. GFP-transgenic Lewis rats (GFP^+^ Lewis rats) were supplied by the National Research Institute for Child Health and Development and bred and maintained in the Laboratory Animal Research Center (Dokkyo Medical University). Lewis rats and GFP^+^ Lewis rats were reared under specific pathogen-free conditions. All protocols were conducted in accordance with the Dokkyo University Regulations for Animal Experiments and with Japanese Government Law (No. 105).

### Experimental design

In a first experiment, Wistar rats received duct-ligation surgery, and the phenotype of infiltrated macrophages into the inflamed pancreas was examined by double immunoenzyme staining for CD68 and other macrophage-related antigens. In a second experiment, Lewis rats received duct-ligation surgery, and the phenotype and proliferation of infiltrated macrophages were examined by three- or four-color immunofluorescence staining. In a third experiment, monocyte-depleted Lewis rats received syngeneic GFP^+^ leukocytes as described below and duct-ligation surgery, and the phenotype and proliferation of infiltrated GFP^+^ monocytes were examined.

General anesthesia was used for injections, surgery, and euthanasia. Anesthesia was provided with isoflurane (Mylan Inc., Tokyo, Japan), administered with an isoflurane vaporizer (SN-487-OT, Shinano Manufacturing, Tokyo, Japan).

### Surgical procedures

Duct-ligation pancreatitis was induced by a surgical procedure as described previously (Yamaguchi et al. 1993). In brief, the rat pancreatic lobes were divided into four segments: splenic, duodenal, gastric, and parabiliary (Richards et al. 1964). In the present study, the pancreatic ducts of the duodenal segment corresponding to the pancreas head in the human were ligated. The operation was performed with a stereomicroscope to avoid compromising the vasculature. The sham-operated rats underwent a midline abdominal incision, and the duodenal loop was pulled caudally, and then, the abdominal wall was closed. In the third experiment, splenectomy was performed just after the duct-ligation surgery.

### GFP^+^ cell transfer to the monocyte-depleted recipients

Inflammatory monocytes are reportedly derived not only from the blood but also from the bone marrow and the spleen (Shand et al. 2014). For the monocyte tracking study, accumulation of recipient monocytes in the inflamed pancreas may compete with that of adoptively transferred GFP^+^ monocytes. In fact, in a preliminary study, we found accumulation of very few GFP^+^ cells when transferred to a duct-ligated recipient without preconditioning.

Accordingly, we tried to deplete recipient monocytes by whole-body sublethal irradiation (Zhou et al. 2008) and splenectomy. The pancreas was protected from irradiation. In this way, monocytes in the blood, bone marrow, and spleen could be depleted considerably without affecting the pancreas by irradiation. In brief, recipient rats were generally anesthetized with intraperitoneal injection of 0.5 ml mixtures of medetomide (75 μg/ml) and midazoram (0.4 mg/ml), and four extremities were fixed with a surgical bandage. The upper abdominal portion was shielded with a lead plate (15 mm thick × 85 mm height × 150 mm width), and the rats received 4 Gy of total-body sublethal X-irradiation (filter: 0.5 mm aluminum + 0.1 mm copper, Hitachi MBR-1505R, Japan). The optimal dose was determined by a preliminary experiment. GFP^+^ cell suspensions as a source of the monocyte lineage were collected from the bone marrow and from the blood of GFP^+^ Lewis rats. The femur, tibia, humerus, and ilium were isolated and the marrow cavity washed out with phosphate-buffered saline (PBS), filtered through a 100-μm nylon mesh, and washed twice in PBS with 0.2 % bovine serum albumin by centrifugation at 280×*g* for 10 min at 4 °C. The white blood cell fraction was isolated with lymphocyte-rat density gradient solution (Cedarlane Laboratories, Ontario, Canada) and washed. These cells were pooled, and ~5 × 10^8^ cells were intravenously injected into the recipient rats after irradiation. At 18 h later, the duct-ligation surgery and splenectomy were performed.

### Tissue preparation

Animals were killed at various intervals after the operation (1, 2, 3, and 4 days for the ligation group, and 2 days for sham-operation controls). Naive rats were also used as the control. One hour prior to kill, recipient rats except for the first experiment received an intravenous injection of a mixture of equivalent moles of BrdU (6 mg/200 g body weight, Sigma-Aldrich Japan, Tokyo) and EdU (5 mg/200 g body weight, Thermo Fisher Scientific, Waltham, MA, USA) in PBS to label proliferating cells. For the first and second experiments, the duct-ligated duodenal segments of the pancreas were excised, and fresh 4-μm-thick cryosections were prepared. For the third experiments using GFP^+^ cells, pancreas tissues were fixed in periodate–lysine–paraformaldehyde and 4-μm cryosections were prepared, as described previously (McLean and Nakane 1974).

### Antibodies and reagents

Mice monoclonal antibodies (mAbs) to rat CD68 (ED1), CD163 (ED2, biotin labeled), CD11b/c (OX42), CD172a (OX41), monomorphic MHCII (OX6 to pan-rat MHCII), and polymorphic MHCII (OX3 to Lewis MHCII) were purchased from AbD Serotec (Kidlington, UK). Alexa Fluor^®^ 488 (Alexa-488)-labeled rabbit anti-GFP IgG was purchased from MBL (Woburn, MA), and rabbit anti-type IV collagen antibody and mice mAb to type IV collagen-like molecules (B12) were kind gifts of Dr. Y. Sado and Dr. T. Ezaki, respectively. CD206 (macrophage mannose receptor) and arginase 1 are reported to be markers of M2 macrophages (Calderon et al. 2015; Hu et al. 2012; Lawrence and Natoli 2011), whereas nitric oxide synthase-2 (NOS2) is an M1 macrophage marker (Lawrence and Natoli 2011). To detect these, we employed rabbit or goat polyclonal antibodies to human CD206, arginase 1, and NOS2 (Santa Cruz Biotechnology, Dallas, TX). Secondary conjugates were Alexa-594-labeled anti-mouse IgG, streptavidin-labeled aminomethylcoumarin (AMCA) (Thermo Fisher Scientific), AMCA-labeled anti-rabbit IgG (Jackson Immunoresearch, West Grove, PA), Alexa-594-labeled goat anti-rabbit IgG (Thermo Fisher Scientific), and Alexa-594-labeled donkey anti-goat IgG (Abcam Plc, Cambridge, UK).

To detect nuclear incorporation of EdU, we used the Click-iT^®^ EdU Alexa-647 Flow kit for imaging (Click-iT kit, Thermo Fisher Scientific).

### Multicolor immunostaining

For the first experiment, cryosections were first stained for CD68 and colored black with chloronaphthol (Sigma Chemical, Co., USA) by an indirect immunoperoxidase method. Sections then were stained for CD163, CD11b/c, or MHCII and colored red with the immunoalkaline phosphatase method (Matsuno et al. 1996). As a result, CD68 antigen was stained as a black dotty granular pattern within the cytoplasm; CD163, MHCII, and CD11b/c (red) are cell membrane-associated molecules and stained as a red homogeneous pattern on the plasma membrane.

For the second and third experiments, multicolor immunofluorescence staining was performed as described previously (Kitazawa et al. 2015). We assigned pseudocolors to each channel to make merged images more comprehensible by maximizing contrast using AxioVision software (Carl Zeiss, Jena, Germany). Generally, pseudocolors of CD68 (green), CD163 (red), EdU (purple), type IV collagen (white), and GFP, CD206, arginase 1, or NOS2 (blue) were combined.

For flow cytometry analysis, GFP^+^ donor cells were stained for Alexa-647-conjugated MHCII (OX3) and either R-phycoerythrin-conjugated CD172a (OX41), CD11b/c, CD161a (3.2.3), CD3 (1F4), CD45R (HIS24) (Biolegend Japan, Tokyo, Japan), or CD163 (self-conjugated with a kit, Dojin, Kumamoto, Japan).

### Definition of interlobular and interacinar areas

The head of the rat pancreas is located in the mesoduodenum, and both ventral and dorsal surfaces are covered by a serosa (visceral peritoneum). Beneath the serosa, a loose connective tissue capsule surrounds each lobule with inward extension. Within the lobules, each acinus and occasional small excretory ducts are also surrounded by delicate connective tissue. We defined the former extralobular region as the interlobular area and the latter intralobular region as the interacinar area. For quantification in the second and third experiments, we mainly counted the cells in the interlobular area because CD68^+^CD163^+^ cells were confined in this location and very few were in the interacinar area.

### Quantification of cellular phenotype and proliferation

For the first experiment, immunostained sections were photomicrographed and the numbers of single- or double-positive cells for CD68 and other markers/mm^2^ of pancreas section counted (mean ± SD, *n* = 3 rats each). For the second and third experiments, multicolor images were captured using an Axioskop2 Plus fluorescent microscope (Carl Zeiss) with an AxioCam MRm camera and AxioVision software (Carl Zeiss) (Kitazawa et al. 2015). Then, annotations were drawn into each image, indicating the single-, double-, or triple-positive cells for CD68, other surface markers, or nuclear EdU. After printing the images with annotations, we calculated the proportions of each phenotype (mean % ± SD, *n* = 3 rats each). Statistical analysis was performed using the Student *t* test.

## Results

### Analysis of Wistar rat pancreas by immunoenzyme staining

**Fig. 1:**
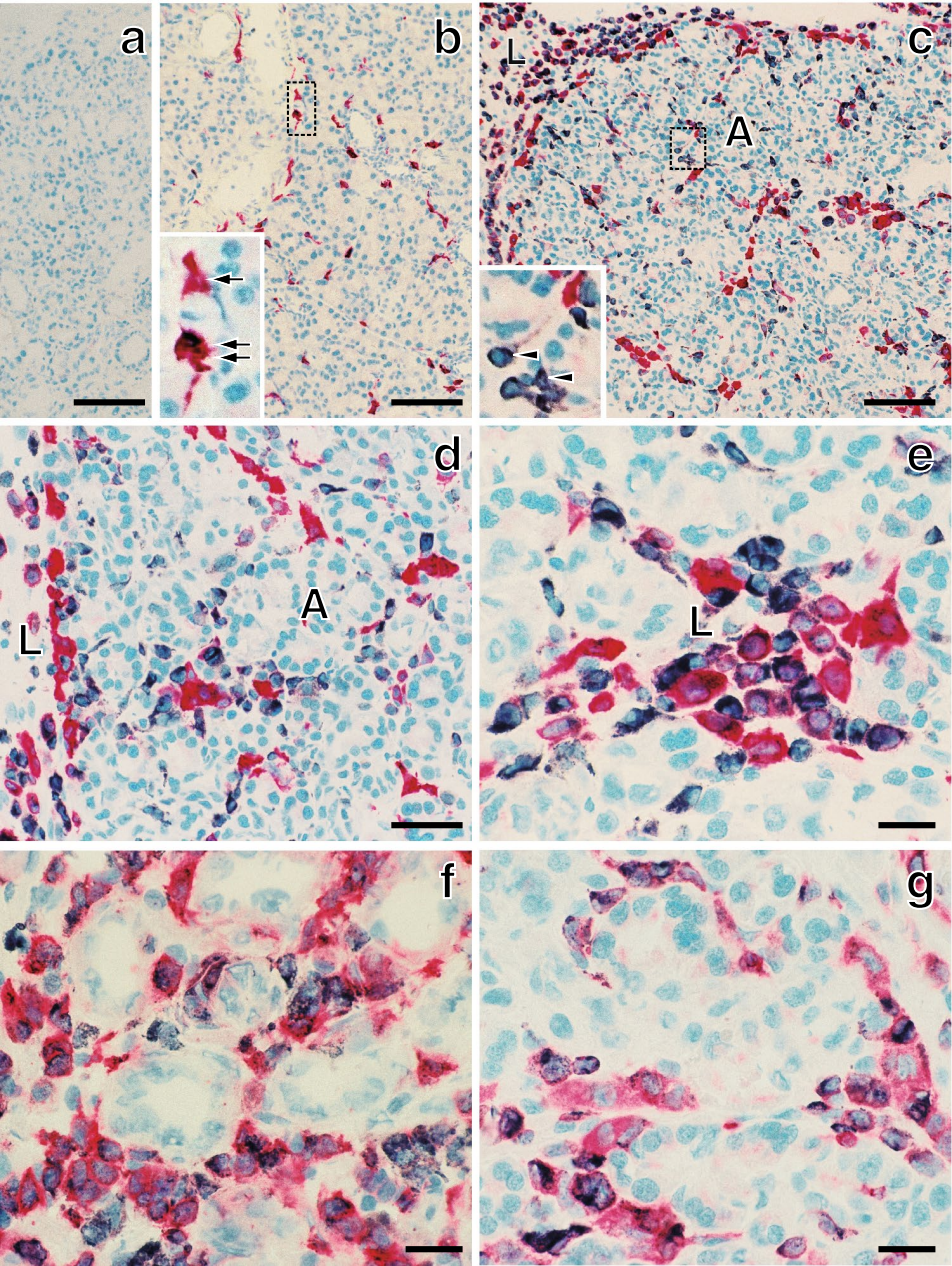
Immunohistological analysis of Wistar rat pancreas. Fresh cryosections are double immunostained for CD68 (*black*) and CD163 (**b**–**e**, *red*), MHCII (**f**, *red*), or CD11b/c (**g**, *red*) and counterstained with hematoxylin. Day 2 (**a**, **c**, **d**, **e**, and **g**) and day 4 (**f**) after duct-ligation surgery. **a** First antibodies are omitted showing no background staining. **b** Untreated control. Single *arrow* and double *arrows* in *inset* of **b** indicate CD68^−^CD163^+^ and CD68^+^CD163^+^ cells, respectively. *Arrowheads* in *inset* of **c** indicate CD68^+^CD163^−^ cells. Note predominant infiltration of CD68^+^CD163^−^ macrophages in the interacinar area (**A**) and CD68^+^CD163^+^ macrophages in the interlobular area (**L**), respectively (**c**–**e**). *Scale bars* **a**–**c** 100 μm; **d** 50 μm; **e**–**g** 20 μm

**Fig. 2:**
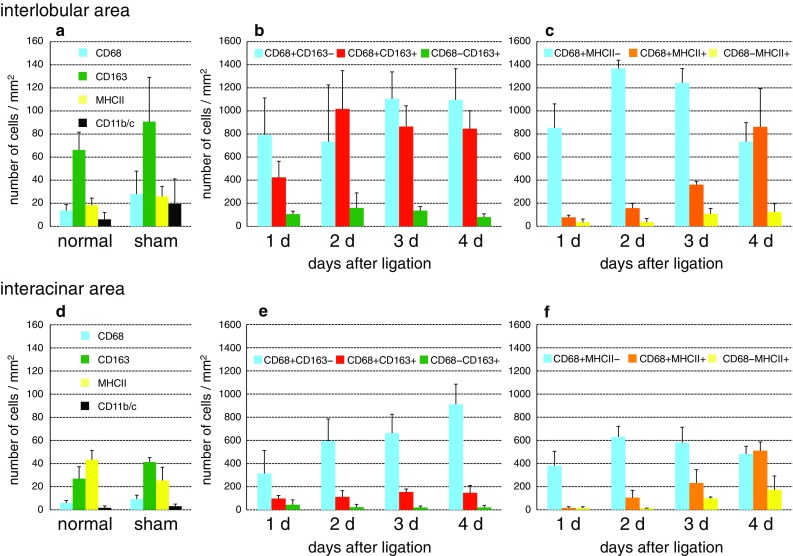
Kinetics of macrophages with different phenotypes in the interlobular (**a**–**c**) and interacinar (**d**–**f**) areas of Wistar rat pancreas. Numbers of single- or double-positive cells for CD68, and other markers/mm^2^ of each area are counted (mean ± SD, *n* = 3 rats each). Note that ~50 % of macrophages are CD68^+^CD163^+^ in the interlobular area (**b**), and the majority of macrophages are CD68^+^CD163^−^ in the interacinar area (**e**). CD68^+^MHCII^+^ cells gradually increased over time in both areas (**c**, **f**). CD68^−^CD163^+^ cells are a minor population, and their number did not change (**b**, **e**)

In naive control rats of the first experiment, the number of CD163^+^ cells in the pancreas was 4–5 times greater than that of the CD68^+^ cells (Figs. 1b, 2a, d). Accordingly, resident macrophages were mostly CD68^−^CD163^+^, as reported in rat skeletal muscle (Honda et al. 1990). Sham-operated rats showed a slight increase in the number of CD68^+^ and CD163^+^ cells (Fig. 2a, d). In addition, highly MHCII-positive (MHCII ^high^) cells with dendritic cytology were located in the peri-ductal areas. These cells were positive for the rat dendritic cell marker CD103 but were CD163^−^ and considered to be dendritic cells (not shown). In this study, these cells were not included.

After duct-ligation surgery, the number of CD68^+^ cells in the inflamed pancreas on day 1 became ~40-fold higher than that observed in the sham-operated rats and increased over time by day 4 (Fig. 2). The double immunostaining revealed that CD68^+^CD163^−^ cells were small and round, resembled monocytes morphologically, and were in the majority in the interacinar area (Figs. 1d, 2e). In contrast, CD68^+^CD163^+^ cells were large and polygonal and accounted for ~50 % in the interlobular area (Figs. 1e, 2b). CD68^−^CD163^+^ cells were a minor population, and their number did not change (Figs. 1d, 2b, e). CD68^+^MHCII^+^ cells gradually increased over time in both areas (Figs. 1f, 2c, f). In addition, most CD68^+^ cells (>90 %) in both areas were positive for CD11b/c^+^ (Fig. 1g, Supplementary Fig. 1).

These findings suggested that there were three macrophage subpopulations: CD68^+^CD163^−^ (monocyte-derived macrophages), CD68^−^CD163^+^ (resident macrophages), and CD68^+^CD163^+^ (macrophages of unknown origin).

### Analysis of Lewis rat pancreas by immunofluorescence staining

**Fig. 3:**
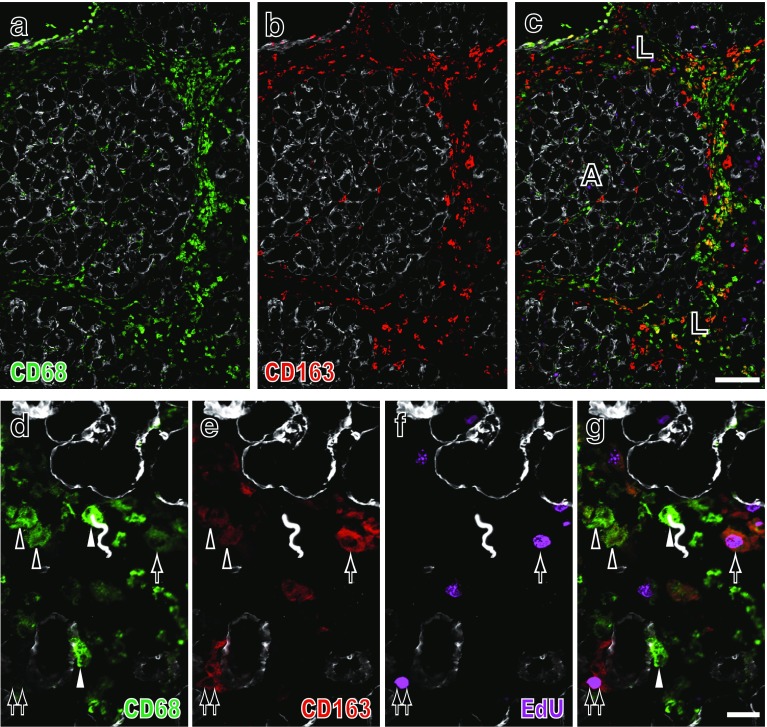
Four-color immunofluorescence staining for CD68 (indirect staining with Alexa-594-conjugated anti-mouse IgG, *green*), CD163 (biotin-labeled mAb plus Alexa-488-conjugated streptavidin, *red*), type IV collagen (indirect staining with AMCA-conjugated anti-rabbit IgG, *blue*), and EdU (Alexa-647-conjugated azide, *purple*). The interlobular area (**L**) and interacinar area (**A**) of Lewis rat pancreas on day 2 after duct-ligation surgery. The same place **a**–**c** and **d**–**g** is photomicrographed for CD68 plus type IV collagen (**a**, **d**), CD163 plus type IV collagen (**b**, **e**), EdU plus type IV collagen (**f**), and merged images (**c**, **g**), respectively. **d**–**g** Four different phenotypes of macrophages in the interlobular area: CD68^+^CD163^−^ (*white arrowhead*), CD68^+^CD163^low^ (*black arrowheads*), CD68^+^CD163^high^ (also EdU^+^, *arrow*), and CD68^−^CD163^+^ (also EdU^+^, *double arrows*) are clearly depicted. Pseudocolors are assigned using AxioVision software. *Scale bars* **c** 100 μm; **g** 20 μm

**Fig. 4:**
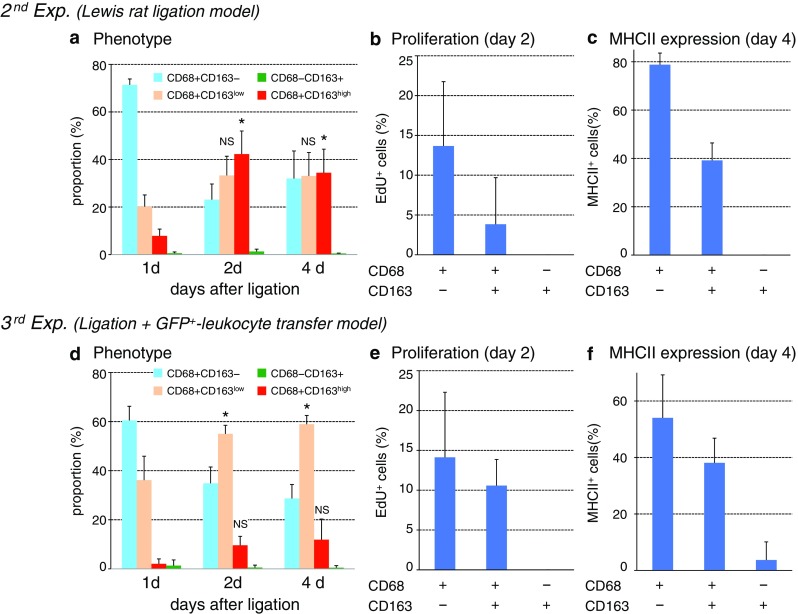
Kinetics of macrophages with different phenotypes in Lewis rat pancreas after duct ligation without (**a**–**c**) or with (**d**–**f**) monocyte depletion plus GFP^+^ leukocyte transfer. The proportions of each phenotype in the interlobular area (mean ± SD, *n* = 3 rats each). Note the significant increase in CD68^+^CD163^high^ cells from day 1 to day 2 (*p < 0.05) and to day 4 (*p < 0.05), respectively (**a**). Both GFP^+^CD68^+^CD163^low^ and GFP^+^CD68^+^CD163^high^ cells showed a slight increase from day 1 and to day 4 (**d**). Approximately 4–14 % of CD68^+^CD163^−^ cells and CD68^+^CD163^+^ cells are EdU^+^ on day 2 in both groups (**b** and **e**). ~80 % of CD68^+^CD163^−^ cells and ~40 % of CD68^+^CD163^+^ cells also expressed MHCII on day 4 in the interlobular area (**c**) with higher proportions than those of GFP cells (**f**). NS, not significant

In the second experiment, four-color immunofluorescence staining of inflamed pancreas clearly depicted cells with four different phenotypes: CD68^+^CD163^−^, CD68^+^CD163^low^ (low positive for CD163), CD68^+^CD163^high^, or CD68^−^CD163^+^ within the interlobular or interacinar areas, which could be identified with type IV collagen immunostaining (Fig. 3). In this way, we confirmed the significant increase in the proportion of CD68^+^CD163^high^ cells from day 1 to day 2 or to day 4 (Fig. 4a, *p* < 0.05), accompanied by a slight increase in CD68^+^CD163^low^ cells in the interlobular area, similar to the first experiment. As for the phenotype of EdU^+^ proliferating cells on day 2, ~15 % of CD68^+^CD163^−^ cells and ~4 % of CD68^+^CD163^+^ cells were EdU^+^ in the interlobular area (Figs. 3d–g, 4b). Approximately 80 % of CD68^+^CD163^−^ cells and ~40 % of CD68^+^CD163^+^ cells expressed MHCII on day 4 in the interlobular area (Fig. 4c).

In contrast, in the interacinar area, CD68^+^ cells mostly did not upregulate CD163 (Figs. 3a–c, 10g). The CD68^−^CD163^+^ cells were stable with no change in cell number and neither proliferated nor upregulated MHCII (Fig. 4b, c). Both findings were the same as those of the first experiment.

### Syngeneic GFP^+^ leukocyte-transfer study to monocyte-depleted Lewis rats

**Fig. 5:**
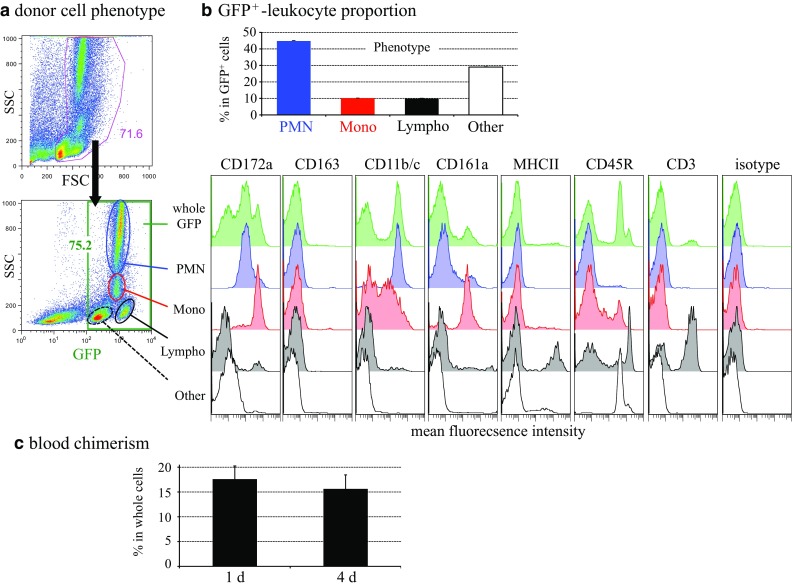
**a** White blood cell fractions prepared from bone marrow and peripheral blood of male GFP-transgenic Lewis rats are analyzed by flow cytometry. Note that ~75 % of whole donor cells are GFP^+^. Monocytes are defined as SSC^mid^ and CD172a^+^CD161a^+^CD11b/c^dull^ cells (encircled in red and depicted by red histogram). Also note that whole GFP^+^ cells as well as monocytes are CD163^−^ and that monocytes are MHCII^−^ before cell transfer. PMN, polymorphonuclear leukocyte; Mono, monocyte; Lympho, lymphocyte. **b** The proportion of GFP^+^ cells and their phenotypes. **c** The proportion of GFP^+^ cells among recipient blood leukocytes 1 or 4 days after transfer

First, we examined the composition of GFP^+^ cells collected from the bone marrow and from the blood of GFP^+^ Lewis rats and the efficiency of the monocyte-depleting regimen in the GFP experiment. By flow cytometry analysis of GFP^+^ donor cells, ~75 % of donor cells were GFP^+^ and 10 % of GFP^+^ cells were monocytes (CD172a^+^, CD163^−^, CD11b/c ^low^, and MHCII^−^) (Fig. 5a). Of note, negligible numbers of total GFP^+^ cells were CD163^+^ (Fig. 5a), demonstrating that the donor monocyte lineage was CD163^−^ before recruitment but upregulated CD163 after infiltration into the interlobular area.

**Fig. 6:**
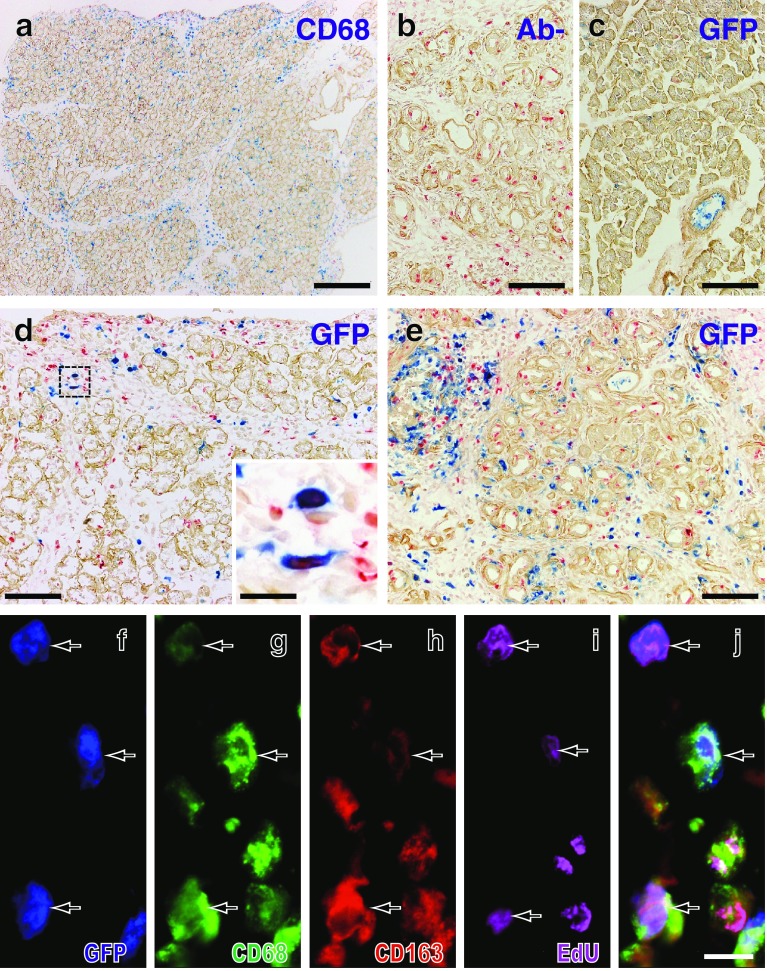
Infiltration of GFP^+^ monocytes in the GFP^+^ leukocyte-transfer experiment. **a**–**e** Three-color immunoenzyme staining for GFP (**c**–**e**, *blue*) or CD68 (**a**, *blue*), type IV collagen (*brown*), and BrdU (*red*). Day 2 (**a** and **d)** and day 4 (**b** and **e**) after duct-ligation surgery and sham-operated control (**c**). **b** Anti-GFP antibody is omitted (Ab−) showing no background GFP staining. Note that GFP^+^ cells increased in number over time but are fewer compared to CD68^+^ cells on day 2 (**d** vs. **a**). A few GFP^+^ cells are BrdU^+^ (*inset* of **d**). **f**–**j** Four-color immunofluorescence staining for CD68 (**g**, indirect staining with Alexa-594-conjugated anti-mouse IgG, *green*), GFP (**f**, Alexa-488–conjugated anti-GFP, *green*), CD163 (**h**, biotin-labeled mAb plus AMCA-conjugated streptavidin, *red*), EdU (**i**, Alexa-647–conjugated azide, *purple*). **j** Merged image of **f**–**i**. *Arrows* indicate either GFP^+^CD68^+^CD163^high^EdU^+^ or GFP^+^CD68^+^CD163^low^EdU^+^ cells. Pseudocolors are assigned using AxioVision software. *Scale bars*: **a** 200 μm; **b**–**e** 100 μm; *inset* of **d** 20 μm; **j** 10 μm

Approximately 15 % of leukocytes in the blood were GFP^+^ during the experiment (Fig. 5c), and CD68^+^ cells outnumbered GFP^+^ cells in the inflamed pancreas (Fig. 6a vs. d). These results indicated that recipient monocytes were only partially depleted by irradiation and splenectomy.

After GFP^+^ leukocyte transfer and duct-ligation surgery, GFP^+^ cells appeared in the inflamed pancreas, and their number increased over time (Fig. 6c–e). The proportion of GFP^+^CD68^+^CD163^low^ cells significantly increased from day 1 to day 2 and to day 4 (Fig. 4d, *p* < 0.05), accompanied by a slight increase in GFP^+^CD68^+^CD163^high^ cells. These results indicated that the CD68^+^ recruited monocytes apparently upregulated CD163 in the interlobular area.

Because GFP^−^CD68^+^ cells might contain non-monocyte macrophages, we examined their phenotype in the interlobular area. The kinetics of GFP^−^CD68^+^CD163^−^, GFP^−^CD68^+^CD163^low^, and GFP^−^CD68^+^CD163^high^ cells (Supplementary Fig. 2) was almost the same as that of GFP^+^ cells (Fig. 4d), suggesting that these GFP^−^ cells were mainly derived from recipient monocytes.

Approximately 15 % of GFP^+^CD68^+^CD163^−^ cells and ~10 % of GFP^+^CD68^+^CD163^+^ cells were EdU^+^ in the interlobular area (Figs. 4e, 6f–j). This result indicated that a portion of recruited monocytes, both CD163^−^ and CD163^+^, were actively proliferating with/without upregulation of CD163. Recruited GFP^+^CD68^+^ monocytes, both CD163^−^ and CD163^+^, upregulated MHCII molecules (Fig. 4f) although to a lesser extent than total CD68^+^CD163^−^ or CD68^+^CD163^+^ cells in the second experiment (Fig. 4c).

### Upregulation of CD206 and arginase 1 by macrophages in Lewis rat pancreas

**Fig. 7:**
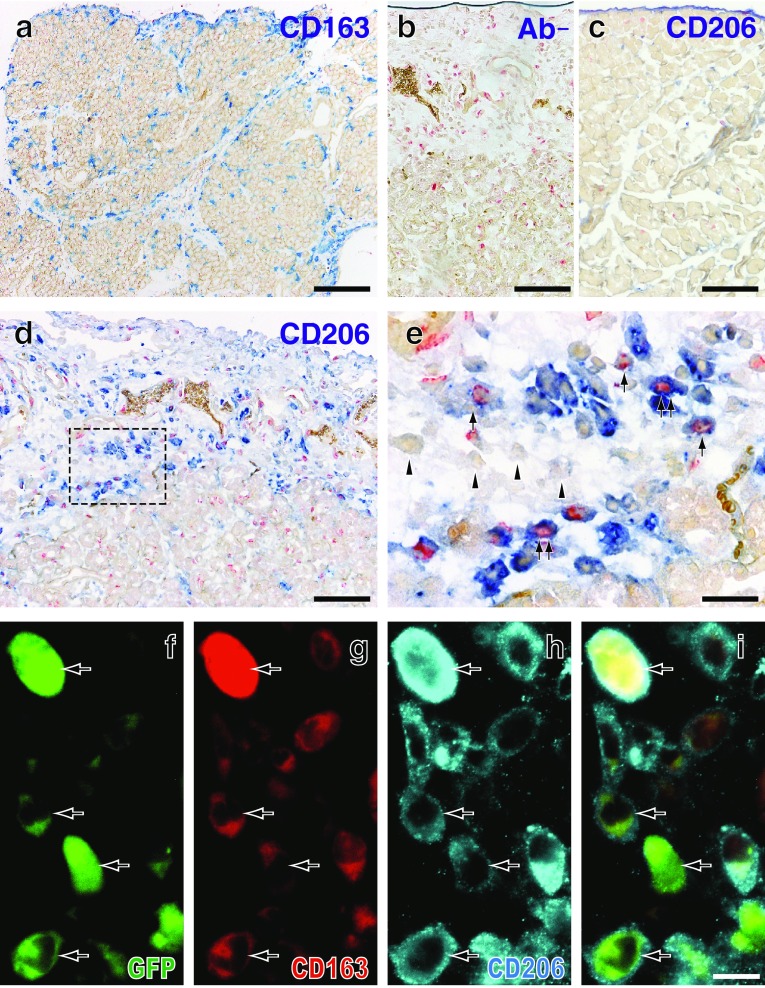
Expression of CD206 by GFP^+^ monocytes in the GFP^+^ leukocyte-transfer experiment. Day 2 (**a**, **b**, **d**, **e**) and day 4 (**f**–**i)** after duct-ligation surgery and sham-operated control (**c**). **a**–**e** Two-color immunoenzyme staining for CD206 (**c**–**e**, *blue*) or CD163 (**a**, *blue*) and BrdU (*red*). **b** Anti-CD206 antibody is omitted (Ab−), showing no background CD206 staining. Note that the number of CD163^+^ cells (**a**) is comparable to that of CD206^+^ cells (**d**) and some CD206^+^ cells are BrdU^+^ (**e**, rectangular area of **d**). **f**–**i** Three-color immunofluorescence staining for CD206 (**g**, Alexa-594-conjugated anti-rabbit IgG, *blue*), GFP (**e**, Alexa-488-conjugated anti-GFP, *green*), CD163 (biotin-labeled mAb plus AMCA-conjugated streptavidin, *red*). **i** Merged image of (**f**–**h**). *Arrows* indicate either GFP^+^CD68^+^CD163^high^ or GFP^+^CD68^+^CD163^low^ cells. Pseudocolors are assigned using AxioVision software. *Scale bars* **a** 200 μm; **b**–**d** 100 μm; **e** 20 μm; **i** 10 μm

In the second and third experiments without/with monocyte depletion, CD206^+^ cells appeared from day 1 and increased in number on day 2, comparable to CD163^+^ cells in the interlobular area (Fig. 7d vs. a). Some of them were actively proliferating on day 2 (BrdU^+^, Fig. 7e). Many GFP^+^CD68^+^CD163^+^ cells were CD206^+^ on day 4 (Fig. 7f–i).

**Fig. 8:**
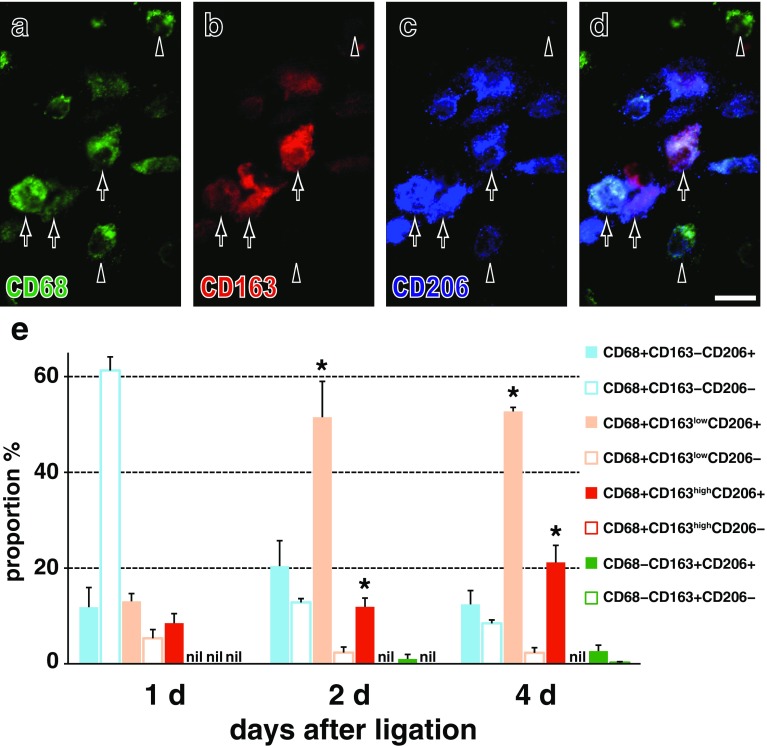
Expression of CD206 by macrophages with different phenotypes in the interlobular area of Lewis rat pancreas on day 4 after duct-ligation surgery. **a**–**d** Three-color immunofluorescence staining for CD206 (**c**, Alexa-594-conjugated anti-rabbit IgG, *blue*), CD68 (**a**, indirect staining with Alexa-488-conjugated anti-mouse IgG, *green*), and CD163 (**b**, indirect staining with AMCA-conjugated streptavidin, *red*). **d** Merged image of **a**–**c**. *Arrows* indicate CD68^+^CD163^high^CD206^+^ or CD68^+^CD163^low^CD206^+^ cells. *Black* *arrowhead* indicates CD68^+^CD163^−^CD206^+^ cell and *white* *arrowhead* CD68^+^CD163^−^CD206^−^ cell. Pseudocolors are assigned using AxioVision software. *Scale bar* **d** 10 μm. **e** Proportions of each phenotype in the interlobular area (mean ± SD, *n* = 3 rats each). Both CD68^+^CD163^low^CD206^+^ cells and CD68^+^CD163^high^CD206^+^ cells significantly increased in number from day 1 to day 2 (*p < 0.05) and to day 4 (*p < 0.05)

**Fig. 9:**
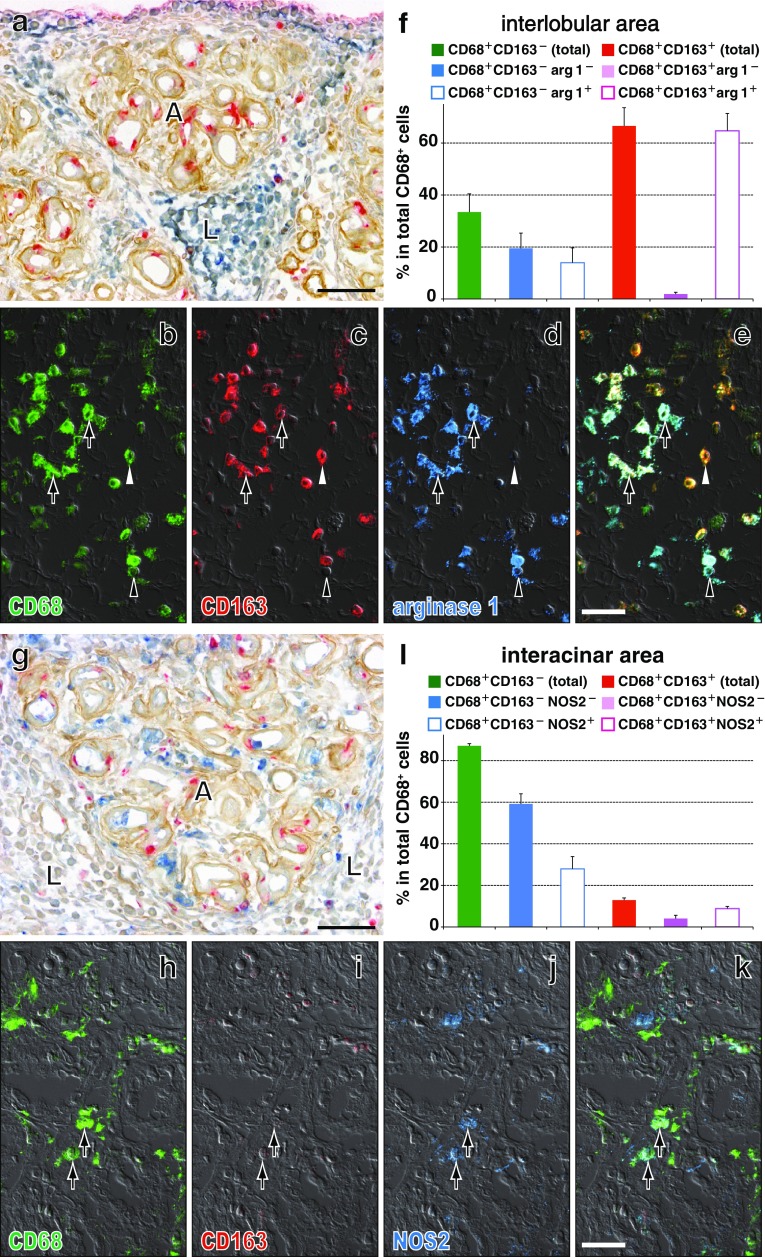
Expression of arginase 1 (**a**–**f**) by macrophages with different phenotypes in the interlobular area and that of NOS2 (**g**–**l**) by macrophages in the interacinar area of Lewis rat pancreas on day 4 after duct-ligation surgery. **a** and **g** Three-color immunoenzyme staining for arginase 1 (**a**, *blue*) or NOS2 (**g**, *blue*), type IV collagen-like molecules (B12, *brown*), and BrdU (*red*). Note preferential localization of arginase 1^+^ cells in the interlobular area (**L**) but not in the interacinar area (**A**). In contrast, NOS2^+^ cells are mainly in the interacinar area (**A**). **b**–**e** and **h**–**k** Three-color immunofluorescence staining for arginase 1 or NOS2 (**d** or **j**, Alexa-594-conjugated anti-rabbit IgG, *blue*), CD68 (**b** and **h**, indirect staining with Alexa-488-conjugated anti-mouse IgG, *green*), and CD163 (**c** and **i**, indirect staining with AMCA-conjugated streptavidin, *red*). **e** and **k** Merged images. Differential interference contrast image is superimposed onto each picture. *Arrows* indicate arginase 1^+^CD68^+^CD163^+^, *black* *arrowhead* is arginase 1^+^CD68^+^CD163^−^, and *white* *arrowhead* is arginase 1^−^CD68^+^CD163^+^ cells. Pseudocolors are assigned using AxioVision software. *Scale bars* **c** 100 μm; **e** 20 μm. **f** and **l** The proportion of each phenotype in the lobular (f) or interacinar areas (l) (mean ± SD, *n* = 3 rats each). Note that most of CD68^+^CD163^+^ cells and ~60 % of CD68^+^CD163^−^ cells are arginase 1^+^ on day 4 in the interlobular area. In contrast, in the interacinar area, CD68^+^CD163^−^ cells partly expressed NOS2

In the second experiment, the proportions of both CD68^+^CD163^low^CD206^+^ cells and CD68^+^CD163^high^CD206^+^ cells significantly increased in the interlobular area over time (Fig. 8a–d, e). Most CD68^+^CD163^+^ cells and ~60 % of CD68^+^CD163^−^ cells were CD206^+^ on day 4 (Fig. 8e). Furthermore, most CD68^+^CD163^+^ cells and ~40 % of CD68^+^CD163^−^ cells expressed arginase 1 (Fig. 9f), but only ~10 % of them expressed NOS2 (not shown).

**Fig. 10:**
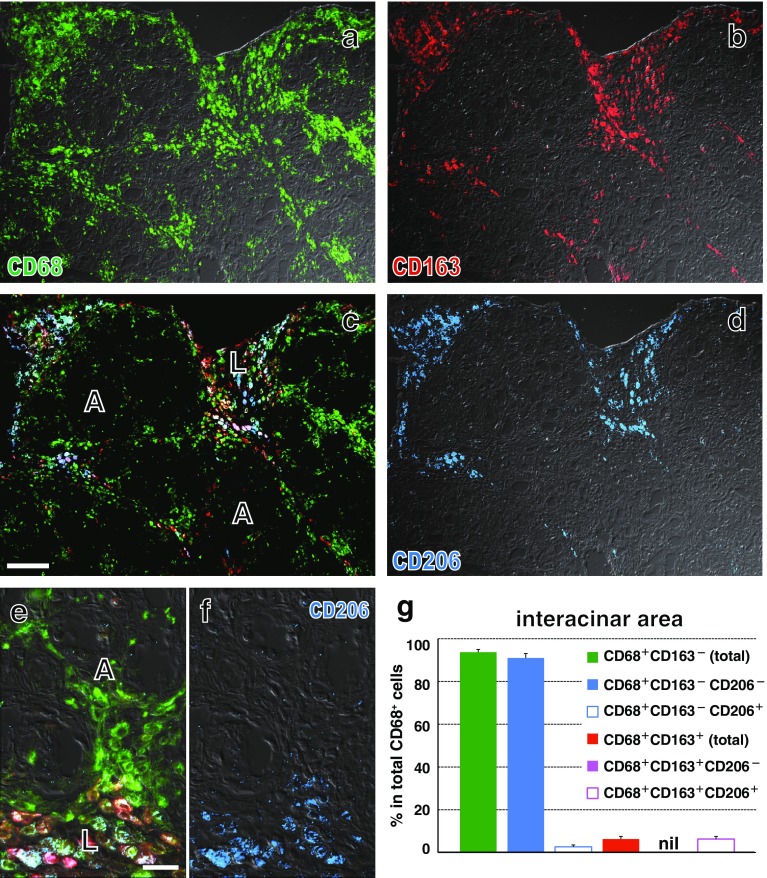
Expression of CD206 by macrophages with different phenotypes in the interacinar area of Lewis rat pancreas on day 4 after duct-ligation surgery. Three-color immunofluorescence staining for CD206 (**d**, **f** Alexa-594-conjugated anti-rabbit IgG, *blue*), CD68 (**a**, indirect staining with Alexa-488-conjugated anti-mouse IgG, *green*), and CD163 (**b**, indirect staining with AMCA-conjugated streptavidin, *red*). **c** and **e** Merged images. A differential interference contrast image is superimposed onto each picture except for **c**. Note that CD68^+^CD163^+^CD206^+^ cells are mostly confined to the interlobular area (**L**) and very few are in the interacinar area (**A**). Pseudocolors are assigned using AxioVision software. *Scale bars* **c** 100 μm; **e** 20 μm. **g** Number of each phenotype/mm^2^ of the interacinar area (mean ± SD, *n* = 3 rats each). Note that there are only a few CD206^+^ and CD163^+^ cells, indicating that CD68^+^ cells upregulated neither CD206 nor CD163

In contrast, in the interacinar area, CD68^+^ cells did not upregulate CD206 (Fig. 10d, g), CD163 (Fig. 10b, g), and arginase 1 (Fig. 9a). Approximately 30 % of CD68^+^CD163^−^ cells expressed NOS2 (Fig. 9l).

## Discussion

### Monocyte recruitment and activation

In the present study, the inflamed pancreas 1–4 d after duct-ligation surgery was infiltrated by mainly macrophages, which were divided into three phenotypes: CD68^+^CD163^−^ round cells, CD68^+^CD163^+^ large polygonal cells, and a few CD68^−^CD163^+^ large fusiform cells. In the GFP^+^ leukocyte-transfer study, monocyte recruitment was confirmed by the presence of GFP^+^CD68^+^ cells in the inflamed pancreas. Although we did not examine this question, we speculate that chemokines such as CCL2 and CCL5 (Haberstroh et al. 2002) might play a role in monocyte recruitment to the inflamed pancreas in the present study.

We found that ~10 % of GFP^+^CD68^+^ monocytes, both CD163^+^ and CD163^−^, became EdU^+^ on day 2 after duct-ligation surgery, indicating active proliferation of recruited monocytes in the inflamed pancreas. In our previous report using Wistar rats (Goto et al. 1993), both CD68^+^ and CD163^+^ cells recorded a higher BrdU labeling index, 20–30 %, possibly because these rats were outbred and reared under conventional housing, which may enhance the proliferating activity of macrophages.

In disease states, monocytes derived from the bone marrow actively proliferate in the periphery (Shand et al. 2014). Concerning growth signals, the pancreatic protease trypsin has been reported to have mitogenic effects (Burger 1970). Macrophage growth factors such as IL-4 (Jenkins et al. 2011) might be also responsible.

### Monocytes in the interlobular area became macrophages with the M2 phenotype

We could directly show that CD68^+^ cells in the interlobular area upregulated CD163 and CD206 over time and expressed arginase 1 on day 4. The absence of CD163^+^ cells among the donor GFP^+^ cells confirmed that the recruited monocyte lineage did upregulate CD163 after infiltration into the interlobular area. CD206 (Calderon et al. 2015; Hu et al. 2012; Lawrence and Natoli 2011), CD163 (Cote et al. 2013; Komohara et al. 2006; Polfliet et al. 2006), and arginase 1 (Lawrence and Natoli 2011) are all M2 macrophage markers, indicating that the recruited monocytes polarized to the M2 phenotype in that location.

To our knowledge, upregulation of CD163 by isolated rat monocytes or recruited rat inflammatory monocytes has been not reported (Cote et al. 2013; Polfliet et al. 2006). Alternative activation of macrophages including upregulation of CD163 or other markers may occur via IL-4 signals or glucocorticoids. Rat peritoneal macrophages upregulate CD163 by glucocorticoids, which could be further enhanced by IL-4 (Polfliet et al. 2006). In mice, there is a solid evidence that thioglycollate-elicited monocyte-derived macrophages could become alternatively activated M2 macrophages after treatment with IL-4 (Jenkins et al. 2011). As for IL-4-producing cells, innate cells such as eosinophils and mast cells are reported to produce this cytokine (Loke et al. 2007). These cells are abundant in the peritoneal cavity, and inflammation of the peritoneal organs including the pancreas may well stimulate IL-4 secretion by these cells (Hu et al. 2012). This cytokine might have penetrated the serosa covering the pancreas and polarized macrophages in the interlobular area to M2 phenotype in the present study.

On the other hand, a portion of CD68^+^CD163^+^ and CD68^+^CD163^−^ cells upregulated MHCII, which is generally considered an M1 marker (Lawrence and Natoli 2011). Because most CD68^+^CD163^+^ cells sustained the M2 phenotype, we consider that MHCII expression at least by CD68^+^CD163^+^ cells may not influence M2 polarization.

Taken together, the results of the present study demonstrated for the first time that rat inflammatory monocytes could upregulate CD163 and CD206 and express arginase 1 and become macrophages with the M2 phenotype in the interlobular area of the inflamed pancreas.

### Monocytes in the interacinar area sustained the M1 phenotype

In contrast to the interlobular area, a majority of CD68^+^ cells in the interacinar area did not upregulate CD163 and were CD206^−^ and arginase 1^−^. Because they partly expressed NOS2 (~30 %), at least some of them might be classically activated M1 macrophages, which promote pro-inflammatory responses. Other NOS2^−^ cells might be monocytes not polarized to M1 or M2 macrophages yet. We previously reported that infiltrating macrophages accumulate in and around acini but not around pancreatic ducts (Yamaguchi et al. 1993). This finding suggests that the acinar portion, but not the ductal system, is the major site of pathology in this pancreatitis model. In this respect, enzyme leakage through the ductoacinar junction into the periacinar space, the gap between the plasma membrane of acinar cells and the subjacent basal lamina, is considered to be secondary to ductal obstruction (Bockman et al. 1971). On the other hand, proliferation of acinar cells and tissue stromal cells continuously occurs up to 28 days (Yamaguchi et al. 1993). Therefore, we consider that the interacinar area retained the pro-inflammatory condition with both destructive and regenerative processes of acinar cells, which might suppress polarization of infiltrated macrophages to the M2 type in this area.

### Resident macrophages

Activation and proliferation of resident macrophages with nematode infection (Jenkins et al. 2011) and skeletal muscle injury (Cote et al. 2013) have been reported. Although resident macrophages are mostly CD68^−^CD163^+^ in the steady state, they might be activated and express CD68 and proliferate in inflammatory conditions. However, in the GFP study, recipient monocytes were only partially depleted by irradiation and splenectomy. Consequently, GFP^−^CD68^+^ cells contained a considerable number of remaining recipient monocytes, and we could not confirm the upregulation of CD68 by resident macrophages in this study.

Because GFP^+^CD68^−^CD163^+^ cells were virtually absent, at least CD68^−^CD163^+^ cells must represent resident macrophages. In the mouse exocrine pancreas, half of resident macrophages are CD206^+^ and considered to be M2 type (Calderon et al. 2015). Therefore, we consider that CD68^−^CD163^+^ cells might represent the M2 type in resident macrophages.

Alternatively, peritoneal macrophages might migrate to the pancreas across the serosa. Half of them are CD68^+^CD163^+^ (Polfliet et al. 2006), and we observed many CD68^+^CD163^+^ cells attached on the serosal surface of the inflamed pancreas (not shown). In a preliminary study, intraperitoneal injection of GFP^+^ peritoneal macrophages resulted in accumulation of a few GFP^+^CD68^+^CD163^+^ cells in the interlobular area, supporting this possibility (unpublished data). Because GFP^+^ monocytes were CD163^−^ before recruitment but did upregulate CD206 and CD163, these peritoneal macrophages may at most contribute in part as infiltrating cells.

### Role of macrophages and conclusion

Infiltrating macrophages were mainly divided into two distinct subpopulations: monocyte-derived macrophages with either the M2 phenotype (GFP^+^CD68^+^CD163^+^CD206^+^arginase 1^+^) in the interlobular area or non-M2 phenotype (GFP^+^CD68^+^CD163^−^CD206^−^arginase 1^−^) in the interacinar area. The latter partly included the NOS2^+^ M1 phenotype. Upregulation of the M2 phenotype by rat inflammatory monocytes was demonstrated for the first time.

M2 phenotype macrophages may promote tissue repair processes in the interlobular area. Although M1 phenotype macrophages were not in the majority in the interacinar area, they might be involved in tissue-destructive processes. Preventing infiltration and M1-type activation of monocytes in the pancreas or polarization of infiltrated monocytes to M2 macrophages in the early periods of pancreatitis might be a therapeutic strategy for severe pancreatitis.

## Electronic supplementary material

Below is the link to the electronic supplementary material.

Supplementary material (PDF 114 kb)
