# Supplementary material for: Expression of area-specific M2-macrophage phenotype by recruited rat monocytes in duct-ligation pancreatitis
Source: Histochem Cell Biol. 2016 Feb 9;145:659–73. doi: 10.1007/s00418-016-1406-y (PMC4848343; doi:10.1007/s00418-016-1406-y)
Supplement: Supplementary file 1 — Supplementary material (PDF 114 kb) [file 418_2016_1406_MOESM1_ESM.pdf]

Supplementary Fig. 1

interlobular area

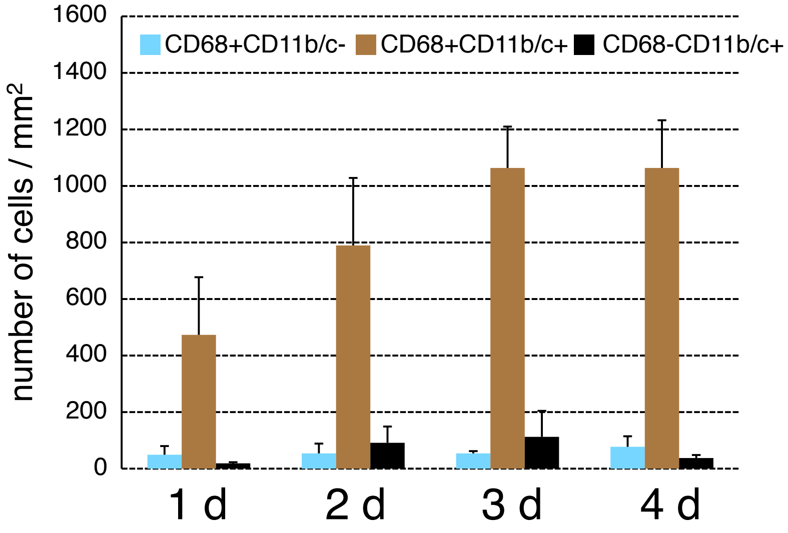

interacinar area

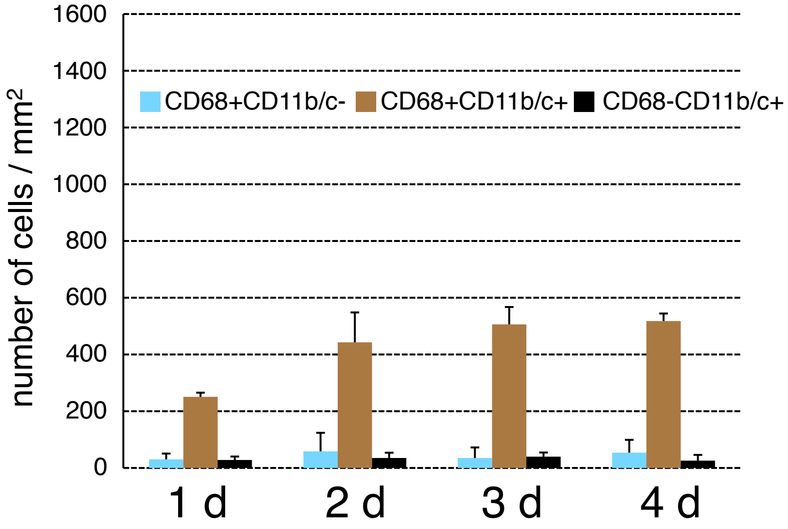

Supplementary Fig. 1

Kinetics of macrophages with different phenotypes in the interlobular and interacinar areas of Wistar rat pancreas. Numbers of single- or double-positive cells for CD68 and CD11b/c/mm^2 of each area were counted (mean ± S.D., n = 3 rats each). Note that most of CD68$^+$ cells (>90%) in both areas were positive for CD11b/c$^+$.

Supplementary Fig. 2

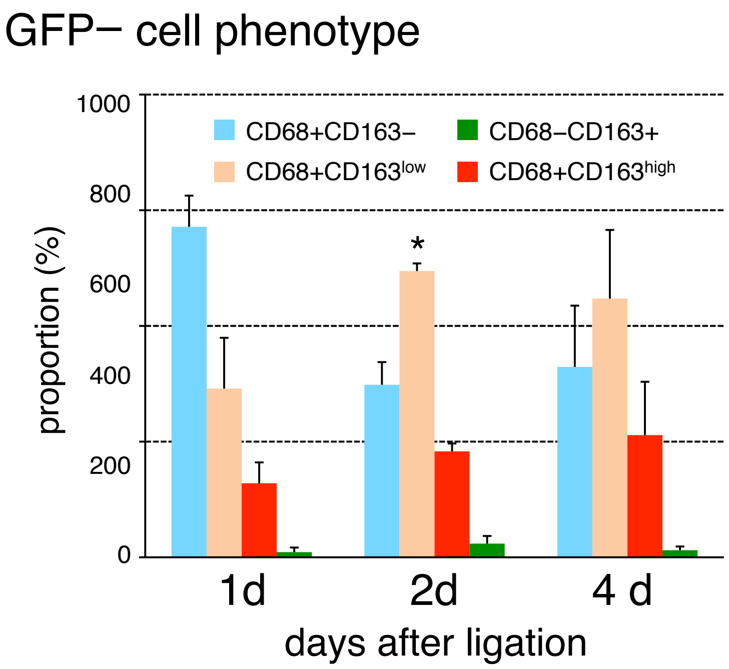

Supplementary Fig. 2

Kinetics of macrophages with different phenotypes in the GFP^{+} leukocyte-transfer experiment. Proportions of GFP^{-}CD68^{+}CD163^{low} cells significantly increased from day 1 to day 2 (*p < 0.05) cells, indicating kinetics similar to that of GFP^{+} cells.
